# High Concentrations of the Antidepressant Amitriptyline Activate and Desensitize the Capsaicin Receptor TRPV1

**DOI:** 10.3390/ph18040560

**Published:** 2025-04-11

**Authors:** Sebastian Pantke, Johanna H. Steinberg, Lucas K. H. Weber, Tabea C. Fricke, Inês Carvalheira Arnaut Pombeiro Stein, George Oprita, Christine Herzog, Andreas Leffler

**Affiliations:** 1 Department of Anesthesiology and Intensive Care Medicine, Hannover Medical School, 30625 Hannover, Germany; pantke.sebastian@mh-hannover.de (S.P.); steinberg.johanna@mh-hannover.de (J.H.S.); weber.lucas@mh-hannover.de (L.K.H.W.); stein.ines@mh-hannover.de (I.C.A.P.S.); georgeoprita13@gmail.com (G.O.); herzog.christine@mh-hannover.de (C.H.); 2 PRACTIS Clinician Scientist Program, Dean’s Office for Academic Career Development, Hannover Medical School, 30625 Hannover, Germany

**Keywords:** amitriptyline, capsaicin, TRPV1, topical treatment, peripheral neuropathic pain, sensory neuron

## Abstract

**Background:** A large number of patients suffer from neuropathic pain, and systemic therapy often remains ineffective while inducing severe side effects. Topical therapy with the TRPV1-agonist capsaicin is an established alternative, and the identification of co-therapeutics that modulate TRPV1 may be a promising approach to reduce the dose of capsaicin while maintaining efficacy. Here, we aimed to determine if the antidepressant amitriptyline displays properties rendering it a potential co-therapeutic agent. **Methods**: We performed patch clamp and calcium imaging experiments on HEK293T cells expressing human (h) TRPV1 as well as on dorsal root ganglion (DRG) neurons from adult mice. **Results**: Amitriptyline induced an increase in intracellular calcium in both HEK293T and mouse DRG neurons expressing TRPV1. Patch clamp experiments revealed a concentration-dependent activation of hTRPV1 by amitriptyline that was also evident in cell-free inside-out patches. When hTRPV1 was fully activated by capsaicin, amitriptyline induced concentration-dependent and partly reversible current inhibition. In contrast, amitriptyline potentiated small responses to capsaicin, heat and protons. We also found that amitriptyline desensitized hTRPV1 to capsaicin. This effect was reduced by the intracellular application of the strong calcium chelator BAPTA. Furthermore, the non-desensitizing mutant hTRPV1-Y672K displayed a reduced amitriptyline-induced desensitization. **Conclusions**: Our data showed that amitriptyline can activate, sensitize, desensitize and even inhibit TRPV1. Together with its property as a strong local anesthetic, our data suggest that amitriptyline may be a promising adjunct to topical capsaicin.

## 1. Introduction

Chronic pain is a burden for patients leading to a significant reduction in quality of life and is causing a high financial strain on health systems [1,2]. Neuropathic pain accounts for about 7–10% of chronic pain [3], and the pharmacological treatment of neuropathic pain remains challenging. As the development of novel agents for the treatment of neuropathic pain suffers from slow translation from basic research into clinical approval, research elucidating the possibility of repurposing approved drugs for pain treatment is warranted. Systemic administration of the tricyclic antidepressant amitriptyline is currently defined as first-line therapy for neuropathic pain [4]. However, this therapy is frequently associated with limited efficacy and adverse side effects [5,6]. While the main “anti-neuropathic” mechanism of systemic amitriptyline seems to be an inhibition of the reuptake of serotonin and noradrenaline [4], amitriptyline can also induce long-lasting local and regional anesthesia by potently inhibiting voltage-gated sodium channels [7,8]. Unfortunately, amitriptyline can induce severe neurotoxicity, prohibiting clinical use as a local anesthetic [9]. Pre-clinical animal studies as well as studies on healthy volunteers or patients suffering from neuropathic pain have also reported analgesia following the topical application of amitriptyline [10,11,12,13,14,15]. However, controlled clinical trials have been ambiguous on whether or not this approach provides significant pain relief [16]. It was also demonstrated that the combined topical application of capsaicin and amitriptyline results in prolonged analgesia in rodents [17]. The capsaicin receptor TRPV1 is a polymodal ion channel expressed in nociceptive somatosensory neurons [18]. As of today, the only approved therapeutic that specifically targets TRPV1 is the 8% capsaicin patch for the topical treatment of peripheral neuropathic pain [19]. The topical treatment of pain is usually devoid of severe side effects, and meanwhile capsaicin, as well as lidocaine patches, are recommended for the treatment of peripheral neuropathic pain [4]. The analgesic effect of capsaicin is mainly due to a TRPV1-mediated neurotoxicity resulting in a reversible degeneration of TRPV1-expressing sensory neurons in the epidermis [20]. For topical lidocaine, the most likely mechanism is an inhibition of voltage-gated sodium channels. High concentrations of lidocaine can also activate TRPV1, and the topical application of lidocaine may even result in a reduction in the epidermal nerve fiber density [21,22]. A joint application of amitriptyline and capsaicin may result in local anesthesia with an immediate onset due to the inhibition of sodium channels by amitriptyline, followed by a long lasting sensory denervation mainly mediated by TRPV1. Considering the rather promiscuous pharmacological profile of amitriptyline, a direct modulation of TRPV1 also needs to be considered. Indeed, amitriptyline was demonstrated to inhibit capsaicin-induced influx in cells expressing TRPV1 [23]. A more recent study suggested that amitriptyline can also induce a rapid increase in intracellular calcium in rodent DRG neurons, presumably by activating the irritant receptor TRPA1 [10]. In this study we aimed to—in detail—characterize the effects of amitriptyline on the human orthologue of TRPV1 in vitro.

## 2. Results

### 2.1. Amitriptyline Activates TRPV1

**Figure 1:**
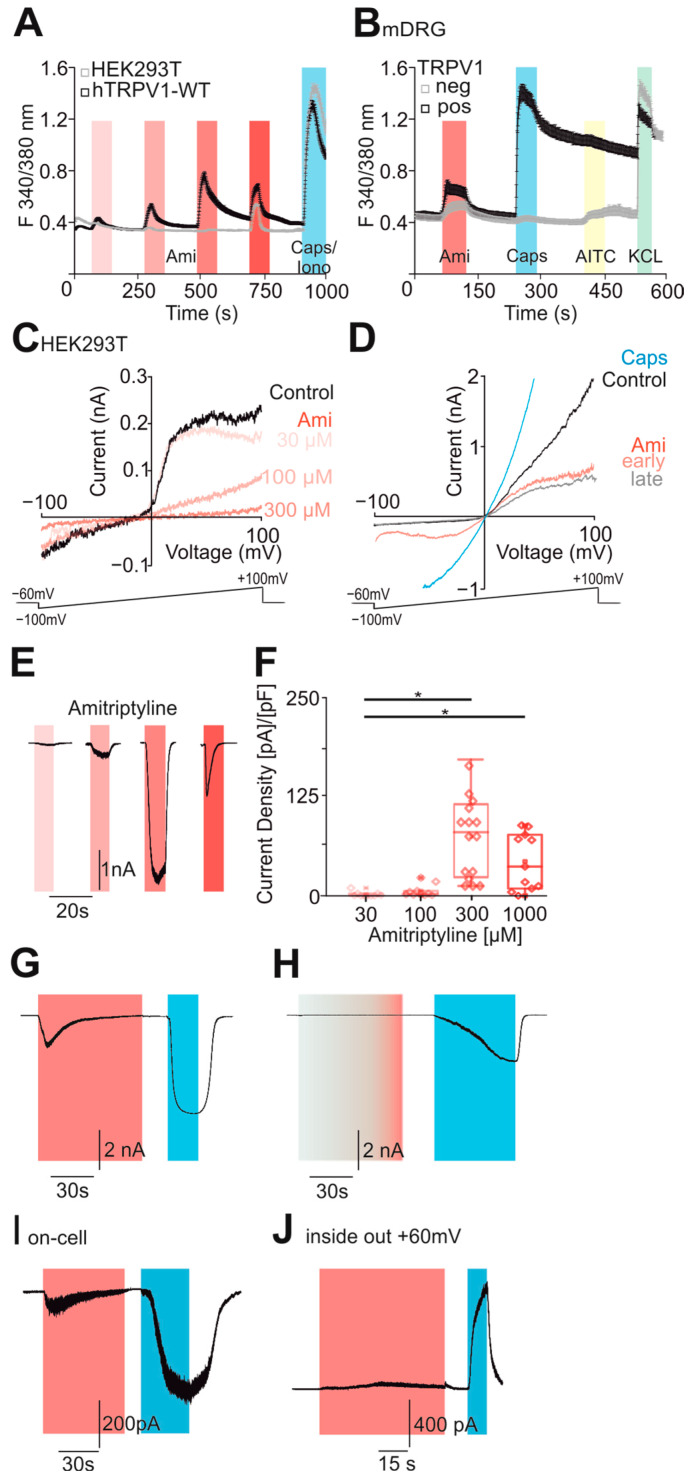
Amitriptyline activates TRPV1. (**A**) hTRPV1-expressing (black curve, *n* = 648) and native HEK293T (grey curve, *n* = 605) cells were investigated by ratiometric calcium imaging. Increasing concentrations of amitriptyline (30, 100, 300, 1000 µM, red bars) are represented by increasing saturation of the bars. Each application was administered for 60 s. A total of 1 µM capsaicin (for TRPV1) and ionomycin (for HEK293T) were administered as positive controls (blue bar). (**B**) In mDRG neurons, 300 µM amitriptyline (red bar), 1 µM capsaicin (blue bar), 100 µM AITC (yellow bar) and KCL (green bar) were applied. The black curve shows the TRPV1-positive DRGs (*n* = 95) and the grey curve shows the TRPV1-negative DRGs (*n* = 196). (**C**,**D**) Additional 500-ms-long voltage ramps from −100 to +100 mV were performed during the application of increasing doses of amitriptyline (30, 100, 300, 1000 µM, red curves, increasing saturation shows the increasing concentration) on (**C**) native HEK293T cells (*n* = 7) or (**D**) cells expressing hTRPV1 (*n* = 7). Early administration of amitriptyline is immediately after application. Late administration is 5 s after application. (**E**) Increasing concentrations of amitriptyline were applied to hTRPV1-expressing HEK293T cells at a holding potential of −60 mV (*n* > 10). Increasing concentrations of amitriptyline (30 µM, *n* = 7; 100 µM, *n* = 7; 300 µM, *n* = 20; 1000 µM, *n* = 11; red bars) are represented by increasing saturation of the bars. Each concentration was applied to a single cell. (**F**) Box diagram with dot plots displaying the current densities of amitriptyline-induced inward currents, induced by the shown concentrations of amitriptyline in Figure 1E in cells expressing hTRPV1 (*n* > 7, one-way ANOVA, Tukey HSD post hoc test). Typical whole-cell recordings show the application of 300 µM amitriptyline for 80 s followed by a positive control of 10 µM capsaicin on ((**G**), *n* = 9) hTRPV1-expressing HEK293T cells without channel blocker ((**H**), *n* = 5) with the TRPV1-specific blocker BCTC. The same protocol was repeated with an on-cell configuration ((**I**), *n* = 8) and an inside-out configuration at the post-positive holding potential ((**J**), *n* = 7). Asterisk (*) denotes *p* < 0.05. The box denotes the 50th percentile (median) as well as the 25th and 75th percentile. The whiskers mark the 5th and 95th percentiles. Data points beyond the whiskers are outliers.

Both hTRPV1-expressing and native HEK293T cells were investigated by the means of ratiometric calcium imaging. Increasing concentrations of amitriptyline were administered for 60 s each. In cells expressing hTRPV1, amitriptyline induced a concentration-dependent (30, 100, 300 and 1000 µM) increase in intracellular calcium (Figure 1A, *n* = 648, black curve). The application of 1000 µM amitriptyline did not result in a further increase in intracellular calcium compared to 300 µM, possibly suggesting a desensitizing effect at high concentrations. However, native HEK293T cells showed an increase in intracellular calcium only with 1000 µM amitriptyline (Figure 1A, *n* = 605, grey curve). In mouse DRG neurons, the application of 300 µM amitriptyline resulted in an increase in intracellular calcium in both TRPA1-positive and TRPV1-negative neurons (Figure 1B, *n* = 196, grey curve), but with somewhat larger responses for TRPV1-positive neurons (Figure 1B, *n* = 95, black curve). The selective TRPV1-agonist capsaicin was used for TRPV1 selection, and the TRPA1 agonist AITC was used for TRPA1 selection. These data suggest that amitriptyline can activate human and mouse TRPV1. Since calcium imaging only detects a change in the intracellular calcium concentration not necessarily generated by TRPV1 channels in the cell membrane, we also examined transfected and naive HEK293T cells using whole-cell patch clamp. During 500-ms-long voltage ramps reaching from −100 to +100 mV, we only observed a concentration-dependent block in the small inward and outward membrane currents in native HEK293T cells (Figure 1C, *n* = 7). However, in TRPV1-expressing cells, amitriptyline induced inward currents that underwent a rather rapid desensitization (Figure 1D, *n* = 7). Outward currents in TRPV1-expressing cells were inhibited by amitriptyline, i.e., an outward rectification known for most TRPV1-agonists was not observed for amitriptyline-induced membrane currents. To examine this finding further, we performed whole-cell patch clamp experiments at a holding potential of −60 mV and applied increasing concentrations of amitriptyline. We observed concentration-dependent activation of inward currents peaking at 300 µM amitriptyline (Figure 1E,F, 30 µM *n* = 7; 100 µM *n* = 7; 300 µM *n* = 20; 1000 µM *n* = 11). In order to verify that amitriptyline indeed activates hTRPV1, we applied 300 µM amitriptyline alone or in combination with the TRPV1-inhibitor BCTC (1 µM). BCTC completely inhibited amitriptyline-induced inward currents, which strongly indicated that amitriptyline activates hTRPV1 expressed in HEK293T (Figure 1G, *n* = 9; Figure 1H, *n* = 5). We also aimed to determine if this was due to direct interaction with the channel or rather attributed to an indirect cytosolic effect. Therefore, we performed on-cell recordings allowing observations of the intact membrane patch and preserved the intracellular conditions and inside-out recordings with small membrane patches detached from the cell. Small amitriptyline-induced membrane currents were observed in the on-cell recordings (Figure 1I, *n* = 8) as well as in the cell-free inside-out patches (Figure 1J, *n* = 7). These data suggest that amitriptyline permeates the cell membrane to activate TRPV1 (on-cell), and that amitriptyline directly activates TRPV1 or at least does not require the cytoskeleton or cytosolic factors.

### 2.2. Amitriptyline Potentiates Capsaicin-, Heat- and Proton-Induced Currents on TRPV1

**Figure 2:**
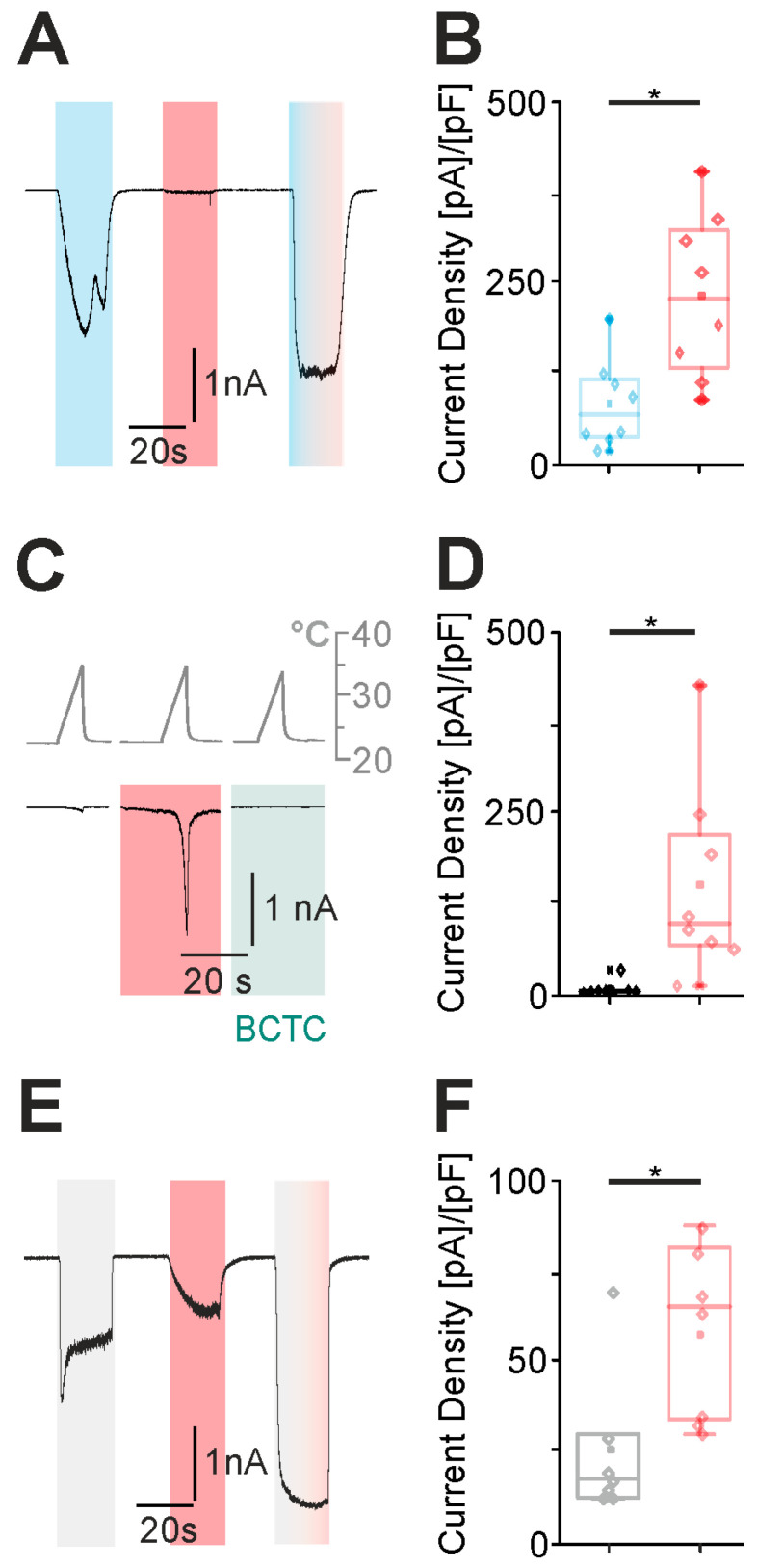
Amitriptyline potentiates capsaicin-, heat- and proton-induced currents on hTRPV1. (**A**,**C**,**E**) Typical patch clamp traces displaying the effect of consecutively and simultaneously applied amitriptyline and 100 µM capsaicin ((**A**), *n* = 8), heat ((**C**), *n* = 8) and protons (pH 6.3) ((**E**), *n* = 7) on HEK293T cells expressing hTRPV1. The TRPV1 agonist was administered first, followed by the single administration of amitriptyline and the subsequent simultaneous administration of both agonists. Each application was carried out for 20 s with subsequent washout. ((**B**,**D**,**F**), paired *t*-test) The corresponding box plots of the current densities of the singular agonist and the simultaneous administration with amitriptyline are shown opposite. Asterisk (*) denotes *p* < 0.05. The box denotes the 50th percentile (median) as well as the 25th and 75th percentile. The whiskers mark the 5th and 95th percentiles. Data points beyond the whiskers are outliers.

Since amitriptyline induced robust membrane currents in TRPV1-expressing cells, we considered if amitriptyline sensitizes TRPV1 activation by other TRPV1 agonists. We applied low concentrations of typical agonists for TRPV1 in combination with 100 µM amitriptyline (9). Indeed, amitriptyline potentiated capsaicin-induced currents (Figure 2A,B, *n* = 8), heat-induced currents (Figure 2C,D, *n* = 8) and proton-induced currents (Figure 2E,F, *n* = 7).

### 2.3. Amitriptyline Blocks and Desensitizes TRPV1

**Figure 3:**
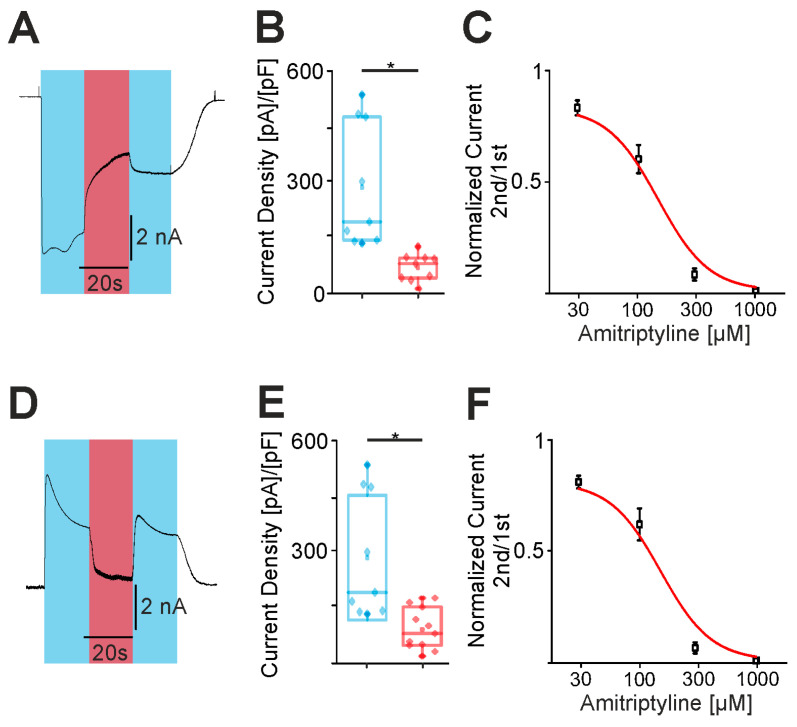
Amitriptyline blocks hTRPV1. (**A**) Amitriptyline-induced (300 µM) block in a 1 µM capsaicin-induced inward current (holding potential −60mV). Amitriptyline was administered during the parallel administration of capsaicin. (*n* = 11). (**B**) Box plots of the current densities of the capsaicin-induced inward current (1 µM, blue bars) and the block by amitriptyline (300 µM, red bars) (*n* = 11, paired *t*-test). (**C**) Representation of the concentration-dependent block by amitriptyline (30, 100, 300, 1000 µM) with a logarithmic regression to determine the IC50 value. ((**D**–**F**), *n* = 9, paired *t*-test) The same protocol was performed as shown above with the positive holding potential (+60 mV) in order to investigate the block in outward currents. Asterisk (*) denotes *p* < 0.05. The box denotes the 50th percentile (median) as well as the 25th and 75th percentile. The whiskers mark the 5th and 95th percentiles. Data points beyond the whiskers are outliers.

**Figure 4:**
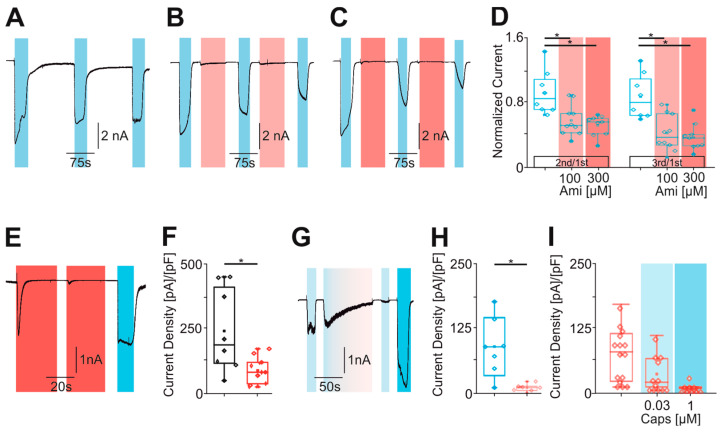
Amitriptyline desensitizes hTRPV1. (**A**–**C**) Typical currents from the repetitive administration of capsaicin with the intermediate administration of 100 µM amitriptyline (**B**) or 300 µM (**C**). (**D**) Box diagram of the normalized currents. The second was normalized to the first capsaicin current and the third to the first capsaicin current. The blue bars represent the capsaicin currents after the administration of amitriptyline. The red boxes represent the application of amitriptyline in between capsaicin administration. The darker the saturation, the higher the concentration of amitriptyline (*n* > 8 each, one-way ANOVA, Tukey HSD post hoc test). (**E**) The repetitive administration of 1 mM amitriptyline (red bars) for 30 s each with the subsequent administration of 10 µM capsaicin (blue bar) (*n* = 9, paired *t*-test). (**F**) The graphs shown are the current densities of 10 µM capsaicin without the prior administration of amitriptyline (black boxes) and after the administration shown in (**E**) (red boxes) (*n* = 9 each, unpaired *t*-test). (**G**) Typical patch clamp trace for a simultaneous administration of 100 µM amitriptyline (red-blue box) with the prior and subsequent administration of 30 nM capsaicin (blue boxes). This was followed by the administration of capsaicin 10 µM (dark blue box). (**H**) The box diagrams show the current densities of the capsaicin currents (30 nM) before and after simultaneous administration (*n* = 7, paired *t*-test). (**I**) Current densities as box diagrams of the amitriptyline-induced currents (300 µM, red boxes). The blue boxes represent the previous application of capsaicin. The darker the saturation, the higher the concentration (*n* > 9 each, one-way ANOVA, Tukey HSD post hoc test). Asterisk (*) denotes *p* < 0.05. The box denotes the 50th percentile (median) as well as the 25th and 75th percentile. The whiskers mark the 5th and 95th percentiles. Data points beyond the whiskers are outliers.

After observing a dose-dependent block in the outward currents in the voltage ramps, we next investigated if amitriptyline is able to block the capsaicin-induced inward and outward currents of TRPV1. Indeed, 300 µM amitriptyline induced a significant inhibition of large inward currents induced by 1 µM capsaicin at −60 mV (Figure 3A–C, *n* = 11). In order to examine if this inhibition was concentration-dependent, we performed further experiments with 30, 100, 300 and 1000 µM amitriptyline. Indeed, we observed a concentration-dependent inhibition with an IC50 value of 146 ± 5 µM (Figure 3C, *n* = 11). Outward currents recorded at +60 mV exhibited an almost complete inhibition by 300 µM amitriptyline (Figure 3D–F, *n* = 9). Experiments with 30, 100, 300 and 1000 µM amitriptyline demonstrated a concentration-dependent inhibition with an IC50 value of 152 ± 6 µM (Figure 3F, *n* = 9). While the inhibition of outward currents was usually rapid and displayed full recovery upon the washout of amitriptyline, the inhibition of capsaicin-induced inward currents was slower and did not recover following the washout of amitriptyline (Figure 3A vs. Figure 3D). This effect on the inward currents might be due to the slower binding kinetics of amitriptyline on inward currents, or to an additional desensitization of TRPV1 and negative membrane potentials. To examine if amitriptyline desensitizes TRPV1, amitriptyline was applied in between three consecutive applications of 1 µM capsaicin in nominal calcium-free extracellular solution (Figure 4A–C). In the control experiments without amitriptyline, inward currents induced by capsaicin only displayed a marginal desensitization (Figure 4A,D, *n* = 8). In contrast, the application of 100 µM (Figure 4B, *n* = 10) or 300 µM (Figure 4C, *n* = 9) amitriptyline induced a concentration-dependent decrease in the amplitudes of capsaicin-induced currents (Figure 4D). In contrast to the capsaicin-induced currents, two consecutive applications of amitriptyline (1000 µM) resulted in inward currents with a rapid inactivation during the application of amitriptyline and a strongly reduced amplitude for the second inward current (Figure 4E, *n* = 9). Importantly, the application of amitriptyline did not result in a total tachyphylaxis by 10 µM capsaicin. To investigate if the response to this high concentration of capsaicin was decreased by amitriptyline as well, we compared the magnitudes of capsaicin-induced currents with or without the application of amitriptyline. The pre-treatment with amitriptyline resulted in a strong reduction in currents induced by 10 µM capsaicin (Figure 4F, *n* = 9 and 8). We next studied the desensitizing effect of a co-application of amitriptyline (100 μM) and capsaicin (30 nM) (Figure 4G, *n* = 7). First, this co-application led to a significant decrease in the current over time. Furthermore, the subsequent inward current induced by capsaicin (30 nM) was significantly reduced compared to the response obtained prior to the co-application (Figure 4H, *n* = 7). Verifying that the TRPV1 channels were still functional following this treatment, the application of 10 µM capsaicin at the end of the experiment induced large currents. We also made the observation that the magnitudes of the amitriptyline-induced currents seem to decline when capsaicin was applied prior to amitriptyline. To quantify this, we compared the current densities of the inward currents induced by 300 µM amitriptyline in cells “pre-treated” with 30 nM or 1 µM capsaicin. The current densities of the amitriptyline-induced currents were indeed significantly reduced by capsaicin (*n* = 12 no capsaicin, *n* = 9; 30 nM capsaicin, *n* = 15 1 µM capsaicin). Although we did not study this effect in detail, it seems possible that it results from a small increase in intracellular calcium due to the activation of TRPV1 channels expressed in the endoplasmic reticulum [24].

### 2.4. Amitriptyline-Induced Desensitization of TRPV1 Is Calcium-Dependent

**Figure 5:**
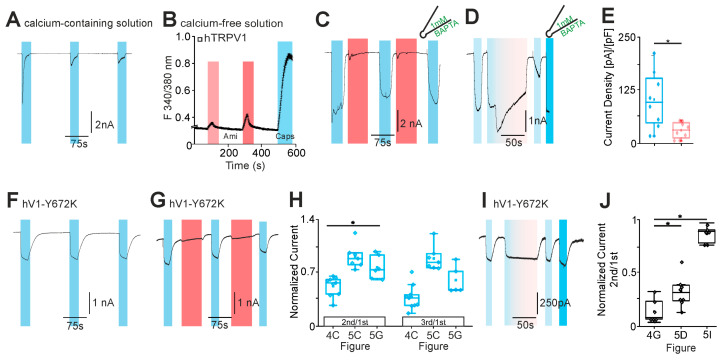
Amitriptyline-induced desensitization of hTRPV1 is calcium-dependent. (**A**) Typical repetitive capsaicin currents (1 µM, blue boxes) in calcium-containing solution (2 mM, *n* = 8). (**B**) Mean increase in F340/380 nm ratio evoked by amitriptyline 100 µM (light red box) and 300 µM (dark red box) in a calcium-free solution. As a positive control for TRPV1, 1 µM capsaicin was administered (blue box) (*n* = 304). (**C**–**E**) Figure 5C has the same protocol as shown in Figure 4C (*n* = 7). Figure 5D (*n* = 8) and E (*n* = 7 and 8, unpaired *t*-test) is the same as shown in Figure 4G,H, with 1 mM BAPTA in the pipette solution. (**F**,**G**) Measurements of the hTRPV1 mutant Y672K with the repetitive administration of 1 µM capsaicin (blue boxes) (**F**) without amitriptyline (*n* = 6) and (**G**) the intermediate administration of 300 µM amitriptyline (red boxes, *n* = 6). (**H**) Box diagrams of the normalized currents. The second was normalized to the first capsaicin current and the third to the first capsaicin current (1 µM, blue boxes). The normalized capsaicin currents of the titled figures are compared (one-way ANOVA, Tukey HSD post hoc test). (**I**) The current shown follows the same protocol as Figure 4G. However, the hTRPV1 mutant Y672K is investigated here (*n* = 6). (**J**) The box plots compare the normalized currents of the capsaicin currents before and after the simultaneous administration of amitriptyline (100 µM). Shown are the normalized currents of the titled figures (one-way ANOVA, Tukey HSD post hoc test). Asterisk (*) denotes *p* < 0.05. The box denotes the 50th percentile (median) as well as the 25th and 75th percentile. The whiskers mark the 5th and 95th percentiles. Data points beyond the whiskers are outliers.

We finally aimed to identify the mechanism responsible for the observed desensitization of TRPV1. As was already reported in several previous studies [25,26], we saw that TRPV1 displays a pronounced desensitization in presence of extracellular calcium (2 mM, Figure 5A, *n* = 8). Since we performed all experiments in nominal calcium-free solutions, we hypothesized that amitriptyline may desensitize TRPV1 by increasing the intracellular calcium. In calcium imaging experiments with calcium-free solution, 300 and 1000 µM amitriptyline induced a concentration-dependent increase in intracellular calcium in hTRPV1-expressing cells (Figure 5B, *n* = 304). Similar to capsaicin, this effect is likely due to an amitriptyline-induced release of calcium from intracellular stores. To determine if an elevation in intracellular calcium accounted for the amitriptyline-desensitization of TRPV1, we performed patch clamp experiments with 1 mM of the strong calcium chelator BAPTA in the pipette solution. The amitriptyline-induced reduction in capsaicin-induced inward currents was strongly abbreviated by BAPTA in the internal solution (Figure 5C, *n* = 8). Furthermore, the strong current decrease observed with the co-application of capsaicin and amitriptyline seemed to be reduced when this experiment was performed with BAPTA in the pipette (Figure 5D, *n* = 8). However, amitriptyline still induced a strong reduction in inward currents induced by 10 µM capsaicin when BAPTA was included in the pipette solution (Figure 5E, *n* = 7 and 8). Instead of adding BAPTA to the cells expressing wildtype hTRPV1, we next employed the mutant hTRPV1-Y672K that was demonstrated to undergo largely no calcium-dependent desensitization [26]. The repeated activation of hTRPV1-Y672K with 1 µM capsaicin in the calcium-free solution resulted in large inward currents devoid of clear desensitization (Figure 5F, *n* = 6). When 1000 µM amitriptyline was applied in between the applications of capsaicin, it only induced a small reduction in current amplitudes (Figure 5G,H, *n* = 6). The co-application of capsaicin and amitriptyline even failed to induce an acute desensitization of hTRPV1-Y672K, and the capsaicin-induced currents were also not reduced following the co-application of both substances (Figure 5I, *n* = 6). In summary, our data suggest that amitriptyline desensitizes TRPV1 by increasing intracellular calcium.

## 3. Discussion

The primary aim of this study was to characterize the effects of amitriptyline on the human orthologue of the capsaicin receptor TRPV1. Our data revealed that high concentrations of amitriptyline modify TRPV1 in a rather complex manner. Depending on either the concentration of amitriptyline or the activation state of TRPV1, amitriptyline can induce channel sensitization, activation, desensitization and inhibition. The relevance of this yet unrecognized pleiotropic property of amitriptyline on TRPV1 is hard to predict, but it may be a rationale for combining amitriptyline and capsaicin to induce topical analgesia.

The capsaicin-, heat- and proton-receptor TRPV1 has been intensively studied as a principal target molecule for the development of novel analgesics. TRPV1 itself, and even more importantly, the population of TRPV1-expressing somatosensory neurons are highly relevant to acute, inflammatory and even neuropathic pain [27]. However, the feasibility of developing selective TRPV1-antagonists into potent systemic analgesics has been questioned. Beside limited analgesic efficacy, TRPV1-antagonists induce transient hyperthermia as an unacceptable side effect [28]. Instead, the possibility to induce long-lasting analgesia by desensitizing or even reversibly ablating sensory neurons by activating TRPV1 can be effective in patients suffering from neuropathic pain [19,20]. Clinical trials also show promising effects following the injection of either a prodrug of capsaicin at the site of surgery for the control of post-operative pain [29], or the ultra-potent TRPV1-agonist resiniferatoxin in joints for the treatment of pain associated with osteoarthritis [30]. As the strong activation of TRPV1 is the principal mechanism for these procedures, associated acute pain is an obvious drawback that can lead to reduced patient satisfaction. Therefore, a co-therapeutic agent that can induce local anesthesia while potentiating the capsaicin-induced activation of TRPV1 seems meaningful. Our data in this report suggest that amitriptyline may be an effective co-therapeutic agent along these lines. Because of its long history as an approved antidepressant and analgesic, the possibility to repurpose amitriptyline for the topical treatment of neuropathic pain therapy may be feasible. With this in mind, there is already substantial literature available on the efficacy of high concentrations (1–10%) of amitriptyline as an off-label topical analgesic [10,15,16]. The high effectiveness of a combination of capsaicin and amitriptyline was only demonstrated in a pre-clinical study on rodents [17]. Therefore, clinical studies are required to further develop this idea. Of note, the local application of the combination of amitriptyline and the NMDA-receptor antagonist ketamine was already investigated in human studies [31,32]. Even if these studies are non-conclusive, this combination seems to be effective in some patients with neuropathic pain. From a mechanistic point of view, we believe that the combination of amitriptyline and capsaicin is likely to be more effective on nociceptive sensory neurons compared to amitriptyline and ketamine. However, similar to the calcium-dependent desensitization of TRPV1 by amitriptyline described in this study, Stepanenko et al. described a similar effect for amitriptyline on NMDA receptors [33]. A fact worth mentioning is also that TRPV1 is a potential therapeutic target for psychiatric diseases. Reyes-Mendes et al. [34] even showed that capsaicin can enhance the anti-depressant effect of a subthreshold dose of amitriptyline in a rodent model for depression. While it is possible that this effect is also a result of the modification of TRPV1 by amitriptyline, we only see the effects of high concentrations (>30 µM) of amitriptyline on the functional properties of TRPV1. As therapeutic systemic plasma levels of amitriptyline are 50 to 300 ng/mL (200–1000 nM, [35]), a relevant effect on TRPV1 seems rather unlikely following the systemic administration of amitriptyline. In the case of topical application for the focal treatment of neuropathic pain, however, up to 10% (~80 mM) amitriptyline is applied without inducing relevant local side-effects [10]. We can only speculate about the effective concentration of amitriptyline that reaches epidermal sensory neurons in this case, but it seems likely that high micromolar concentrations may build up in the epidermis. This note is supported by the rather effective local anesthesia that locally applied amitriptyline can induce [10]. The effective inhibition of C- and A-fiber excitability requires 100 µM amitriptyline, and a relevant block in neuronal sodium channels was reported to be induced by >10 µM amitriptyline [10]. So even if TRPV1 does not seem to be a high-affinity target of amitriptyline, it seems justified to assume that TRPV1 channels expressed in epidermal sensory fibers are modulated by topically applied amitriptyline.

While Colvin et. al. postulated that amitriptyline may enter nociceptors via TRPV1 in order to inhibit sodium channels [17], we can demonstrate a direct effect on TRPV1. While the inhibition of TRPV1 is likely to be explained by a pore block shown for several other substances including lidocaine [21], the desensitization is due to a release of calcium from intracellular stores. We did not further examine the intracellular mechanisms accounting for this release of calcium from intracellular stores, but previous reports suggested that this effect is mediated by inositol 1,4,5-trisphosphate-sensitive calcium stores [36,37]. The exact mechanism accounting for the activation of TRPV1 by amitriptyline also remains unclear, but our data indicate a direct interaction of amitriptyline with TRPV1.

Taken together, our data demonstrated a modification of TRPV1 by amitriptyline with the potential for clinical implications.

## 4. Materials and Methods

### 4.1. Chemicals

Chemicals were obtained and dissolved as follows: Amitriptyline (Amitriptyline hydrochloride) was purchased from Sigma-Aldrich (Taufkirchen, Germany), was stored at +4 °C and dissolved to a concentration of 1 M in external solution before every use. BCTC (N-(4-tertiarybutylphenyl)-4-(3-chloropyridin-2-yl) and tetrahydropyrazine-1(2H)-carbox-amide) was purchased from Biotrend (Wangen/Zurich, Switzerland), diluted in DMSO and stored at +4 °C. Capsaicin from HelloBio (Bristol, UK) was diluted in ethanol and stored at +4 °C. BAPTA (N,N′-[1,2-ethanediylbis(oxy-2,1-phenylene)]bis[N-[2-[(acetyloxy)methoxy]-2-oxoethyl]-1,1′-bis[(acetyloxy)methyl] ester-glycine) was obtained from Cayman Chemical (Ann Arbor, MI, USA), stored at −20 °C and dissolved in an internal solution. Fura-2-AM was dissolved in DMSO to 1 mM and was purchased from Biotrend (Cologne, Germany).

### 4.2. cDNA and Cell Culture

Human embryonic kidney 293 (HEK293) T cells were cultured in Dulbecco’s modified Eagle’s medium (DMEM, Darmstadt, Germany) supplemented with 10% fetal bovine serum (Biochrom, Berlin, Germany) and 1% penicillin/streptomycin under standard cell culture conditions (5% CO_2_ at 37 °C). Plasmids for human hTRPV1 and the mutant hTRPV1-Y672K were transfected into HEK293T cells using jetPEI (VWR, Darmstadt, Germany). Cells were detached 24 h post-transfection using phosphate-buffered saline (PBS, Lonza, Cologne, Germany).

### 4.3. Mutant Design and Generation

The calcium-insensitive mutant hTRPV1-Y672K was generated using site-directed mutagenesis with the QuikChange Lightning Kit (Agilent, Waldbronn, Germany) according to the manufacturer’s instructions. Sequencing confirmed the intended amino acid exchange and ensured that no unintended mutations were introduced.

### 4.4. Animals

Dorsal root ganglia (DRG) were prepared from C57Bl/6 WT mice as previously described [21]. Mice were anesthetized with isoflurane and decapitated. DRG neurons were collected from all spinal column levels and transferred to DMEM. Ganglia were digested in DMEM containing 1 mg/mL collagenase and 0.5 mg/mL protease (all from Sigma-Aldrich, Taufkirchen, Germany) for 45 min, then separated with Pasteur pipettes. Isolated cells were plated on poly-L-lysine-coated (0.1 mg/mL, Sigma-Aldrich) coverslips and cultured in TNB 100 medium supplemented with TNB 100 lipid protein complex, penicillin/streptomycin (100 U/mL) and mouse NGF (100 ng/mL, Almone Laboratories, Tel Aviv, Israel) for 24 h.

Institutional Review Board Statement: Tissue removal from adult mice was approved by the Animal Protection Officer of Hannover Medical School [§4-Anzeige, Nummer 3741, Approval date 1 January 2024]. Animal studies comply with the ARRIVE guidelines [38] and adhere to the recommendations of the British Journal of Pharmacology [39].

### 4.5. Patch Clamp

Recordings were conducted at room temperature using an EPC10 USB HEKA amplifier (HEKA Elektronik, Lambrecht, Germany). Signals were low-pass filtered at 1 kHz and sampled at 2 to 10 kHz. Patch pipettes, with resistances ranging from 2.0 to 5.0 MΩ, were pulled from borosilicate glass tubes (TW150TF-8; World Precision Instruments, Berlin, Germany). Internal solution: KCl 140 mM, MgCl_2_ 2 mM, EGTA 5 mM and HEPES 10 mM (pH 7.4 adjusted with KOH). External solution: NaCl 140 mM, KCl 5 mM, MgCl_2_ 2 mM, EGTA 5 mM, HEPES 10 mM and glucose 10 mM (pH 7.4 adjusted with NaOH). Calcium was omitted to prevent desensitization. For solution application, a gravity-driven multi-barrel perfusion system was employed. The on-cell configuration was established by forming a high-resistance gigaseal by gently approaching the cell membrane with the pipette tip under slight suction. This configuration maintained the intact membrane patch, preserving intracellular conditions and allowing extracellular solution manipulation. Cells were held at −60 mV during these recordings. The inside-out configuration was achieved by retracting the pipette from the cell after gigaseal formation, detaching the membrane patch. The intracellular side of the membrane was exposed to the external solution, enabling controlled intracellular manipulation. Cells were held at +60 mV. Voltage ramps, spanning from −100 mV to +100 mV, were applied over 500 ms in the whole-cell and on-cell configurations. To heat the solution from room temperature to ~45 °C within 10 s, a current was applied through an insulated copper wire around the capillary tip of the application system’s output. The solution temperature was measured at the capillary tip using a miniature thermocouple placed near the cells. Data acquisition and offline analyses were performed using Patchmaster/Fitmaster (HEKA Elektronik, Lambrecht, Germany) and Origin 8.5.1 (OriginLab, Northampton, MA, USA). A fluorescent light source (HXP 120, LEJ Lighting & Electronics Jena, Jena, Germany) with appropriate filter sets (Chroma Technology GmbH, Olching, Germany) was used to illuminate the cells with UVA light.

### 4.6. Ratiometric Imaging

For ratiometric calcium imaging, cells were seeded on coverslips at least 4 h before measurement and stained for 45 min with 4 µM Fura-2-AM and 0.02% Pluronic. After washing, coverslips were placed on an inverted microscope (Axio Observer D1, Zeiss, Jena, Germany). Cells were illuminated with UV light at 340 nm and 380 nm using a light source (HXP120, LEJ Lighting & Electronics, Jena, Germany), an LEP filter wheel (Ludl Electronic Products Ltd., Hawthorne, NY, USA) and appropriate filter sets (Chroma Technology GmbH, Olching, Germany). Images were acquired with a CCD Camera (Cool SNAP EZ, Photometrics, Puchheim, Germany). Data were recorded using VisiView 2.1.1 software (Visitron Systems GmbH, Puchheim, Germany). The standard solution contained: NaCl 145 mM, KCl 5 mM, CaCl_2_ 1.25 mM, MgCl_2_ 1 mM, glucose 10 mM and HEPES 10 mM (pH 7.4). Cells without functional hTRPV1 expression were excluded. Capsaicin and the Ca^2+^-ionophore ionomycin were used to verify TRPV1 expression. Results are presented as mean (±SEM) of the F340/380 nm ratio.

### 4.7. Statistical Analysis

Data are shown as mean ± S.E.M. Sample sizes represent the number of measured cells. Imaging data were collected from at least three experiments conducted on different days. Patch clamp data were collected from experiments performed on at least two separate days. Related group comparisons used the paired *t*-test, while unrelated groups used the unpaired *t*-test. ANOVA with Tukey HSD post hoc test was used for the comparisons of more than two groups. Calculations were performed using Origin 8.5.1 (OriginLab, Northampton, MA, USA). The significance level was set at *p* < 0.05.

## 5. Conclusions

Our data demonstrated a complex and bimodal modulation of TRPV1 by amitriptyline. The exact mechanisms for channel activation of TRPV1 by amitriptyline remain to be defined, but our data suggest that a direct interaction of amitriptyline with TRPV1 accounts for the effect. TRPV1 channel inhibition is likely due to an immediate pore block as well as to desensitization triggered by a release of calcium from intracellular stores.

## Data Availability

The raw data supporting the conclusions of this article will be made available by the authors on request.

## Abbreviations

The following abbreviations are used in this manuscript:

BAPTA	(N,N′-[1,2-ethanediylbis(oxy-2,1-phenylene)]bis[N-[2-[(acetyloxy)methoxy]-2-oxoethyl]-1,1′-bis[(acetyloxy)methyl] ester-glycine)
BCTC	4-(3-Chloro-2-pyridinyl)-N-[4-(1,1-dimethylethyl)phenyl] 1piperazinecarboxamide
DMEM	Dulbecco’s modified Eagle medium
DMSO	Dimethylsulfoxide
DRG	Dorsal Root Ganglion
Fura-2-AM	Fura-2-pentakis-(acetoxymethyl)-ester
HEK293T	Human Embryonic Kidney 293T
NeuPSIG	Neuropathic Pain Special Interest Group of the International Association for the Study of Pain
NMDA	N-Methyl-D-Aspartate
TRPV1	Transient Receptor Potential Vanilloid 1
